# Arthroscopic reduction of a locked patellar dislocation: a new less invasive technique

**DOI:** 10.1007/s00167-018-4959-6

**Published:** 2018-05-11

**Authors:** João Teixeira, Carlo Gamba, Jan Ophuis, Geert A. Buijze, Gino M. M. J. Kerkhoffs

**Affiliations:** 1 Department of Orthopaedic and Traumatology, Centro Hospitalar de Entre o Douro e Vouga, Santa Maria da Feira, Portugal; 2 Department of Orthopaedic and Traumatology, Hospital de la Santa Creu i Sant Pau, UAB, Barcelona, Spain; 3 Department of Orthopaedic Surgery, Orthopaedic Research Center Amsterdam, Academic Medical Center, PO Box 22660, 1100 DD Amsterdam, The Netherlands; 4 Academic Center for Evidence based Sports medicine (ACES), Amsterdam, The Netherlands; 5 Amsterdam Collaboration for Health and Safety in Sports (ACHSS), Amsterdam, The Netherlands

**Keywords:** Patellar dislocation, Lateral retinaculum, Patella, Arthroscopy, Sports trauma, Arthroscopic patellar reduction, Minimally invasive surgery

## Abstract

Patellar dislocation is a condition that is often reduced by itself or through closed manipulation from a trained professional. In this case of a traumatic lateral patellar dislocation, the patella was caught through the rupture in the lateral retinaculum, as is seen in Boutonniere-like lesions. Reduction of the dislocated patella was obtained by arthroscopic reduction.

*Level of evidence* V.

## Introduction

About 3% of all knee injuries are acute patellar dislocations [8]. The average incidence is 29 cases per 100,000 in the 10- to 17-year-old age group [8]. Therefore, it is a relatively common injury in children and adults that demands emergency care.

Patients often report a flexion valgus movement of the knee, feeling the patella dislocate. If spontaneous reduction by extending the knee is not possible, extension of the flexed knee with pressure applied to the lateral margin of the patella result may result in reduction [10]. The patella is predominantly dislocated laterally.

A total of 16 cases were identified in literature where the patella remains locked and proves irreducible by closed methods [10]. This type of dislocation was first described by Cooper in 1844 and is usually associated with patellar rotation around the longitudinal axis [3]. This type of dislocation can be classified as intra-articular in cases where the patella is locked within the femoral condyles, or extra-articular when the dislocated patella is wedged against the condyle, usually on the lateral side of the lateral femoral condyle [20, 23].

An overview of cases where patients have needed surgical intervention with open reduction or simple closed reduction after sedation is given in Table 1. To the best of our knowledge, there is no report of an arthroscopic reduction procedure. The aims of this article are to describe a case report of an arthroscopic patellar reduction in a young patient with a locked patellar dislocation associated to a direct high-energy trauma and to give a short overview of the currently available literature on irreducible patellar dislocations.

**Table 1:** Literature review of all reported cases of locked patellar dislocation

Authors	Year	Age	Sex	Traumatic mechanism	Cause of irreducibility	Additional images	Reduction method	Additional treatment	Immobilization
Inman [14]	1941	43	M	Yes	Long axis rotation	Different tangential views	Open	Medial repair	2 weeks
Moed [18]	1982	18	M	No	Long axis rotation	–	Closed	–	3 weeks
Benjamin [2]	1984	38	F	No	Long axis rotation	No	Open	Soft tissue reconstruction	3 weeks
Corso [4]	1990	16	M	Yes	Long axis rotation	No	Open	Medial repair and plication	6 weeks
Hackl [11]	1999	53	F	Yes	Lateral femoral condyle impaction	CT scan	Open	MPFL anchor re-attachment + lateral retinaculum release	No. Early passive ROM
Gorczya [9]	2000	13	M	Yes	Long axis rotation	No	Open	Medial repair	No
ElMaraghy [6]	2002	30	F	Yes	Long axis rotation	No	Open	Medial repair	5 weeks full weight bearing allowed
Phaltankar [21]	2002	66	M	No	Lateral femoral impaction	MRI	Open	Total knee arthroplasty	No
Sherman [22]	2004	28	M	Yes	Long axis rotation	No	Closed	No	4 weeks
Abdelhalim [1]	2007	8	M	Yes	Long axis internal rotation	–	Closed	–	4 weeks
Feibel [7]	2007	66	F	Yes	Lateral femoral impaction	CT	Open	No	No early mobilization
Huang [13]	2008	12	M	Yes	Long axis rotation	No	Closed	No	3 weeks
Michels [17]	2008	16	F	No	Long axis internal rotation	CT	Open	Lateral release–medial plication	2 weeks
C. Yang [24]	2010	19	M	Yes	Long axis internal rotation	No	Closed	No	6 weeks
Louw [15]	2012	17	F	No	Lateral femoral impaction	CT	Open	Medial plication + coverage lateral defect with iliotibial band flap	2 weeks
Lowe [16]	2012	50	M	Yes	Lateral femoral impaction	–	Open	Medial repair	2 weeks
Yerimah [25]	2013	21	M	Yes	Lateral femoral impaction	–	Open	Medial repair	4 weeks
Grewal [10]	2014	32	F	Yes	Lateral femoral impaction vs soft tissue	CT	Closed		2 weeks
Devgan [5]	2016	14	M	No	Long axis internal rotation	No	Open	Lateral release–medial plication	No. WB at 2 weeks
Higgins [12]	2016	32	M	No	Long axis external rotation	–	Open	Medial repair	5 days

## Case report

A 23-year-old male was presented at the emergency department following a direct trauma to the knee during a football match when another player fell on his extended knee. An evident deformity was present in the knee suggesting a lateral displacement of patella. The main symptoms were pain and limited range of motion; however, there was no neurovascular damage. The knee was locked in almost complete extension.

**Fig. 1:**
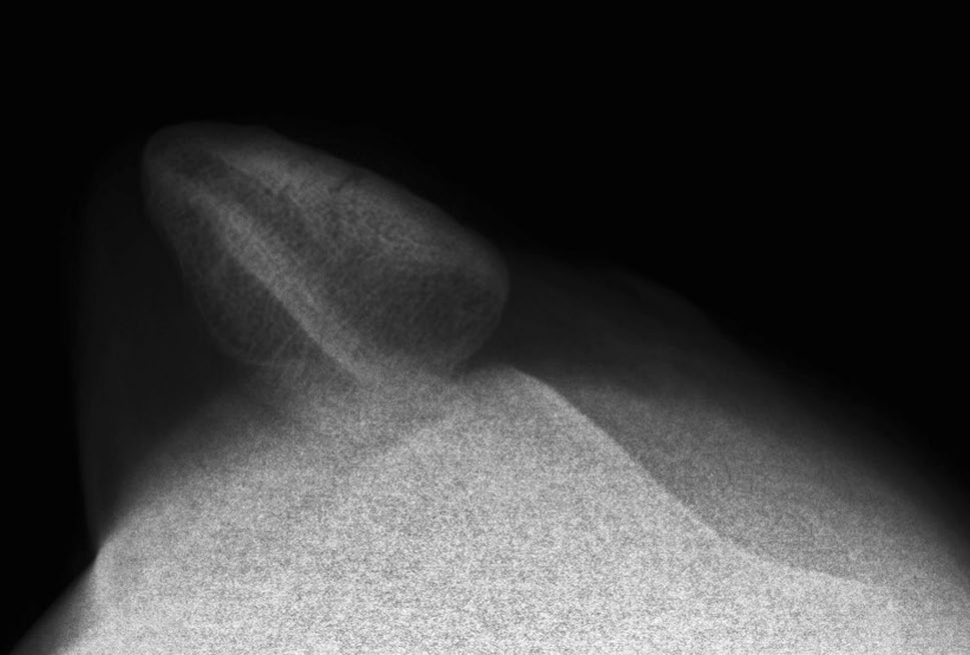
Right knee radiograph: axial view confirming the lateral dislocation without rotation in the longitudinal axis

Radiological examination of the knee was conducted confirming a lateral displaced patella without any other apparent lesion. It also showed that the patella was not laterally rotated, as is common in most of the cases of patellar dislocation [9], but maintained a correct coronal alignment (Fig. 1).

A closed reduction with hyperextension and manipulation of the patella towards medial was attempted first in the emergency department, followed by a second attempt under general anaesthesia in the operation room; both attempts were unsuccessful.

**Fig. 2:**
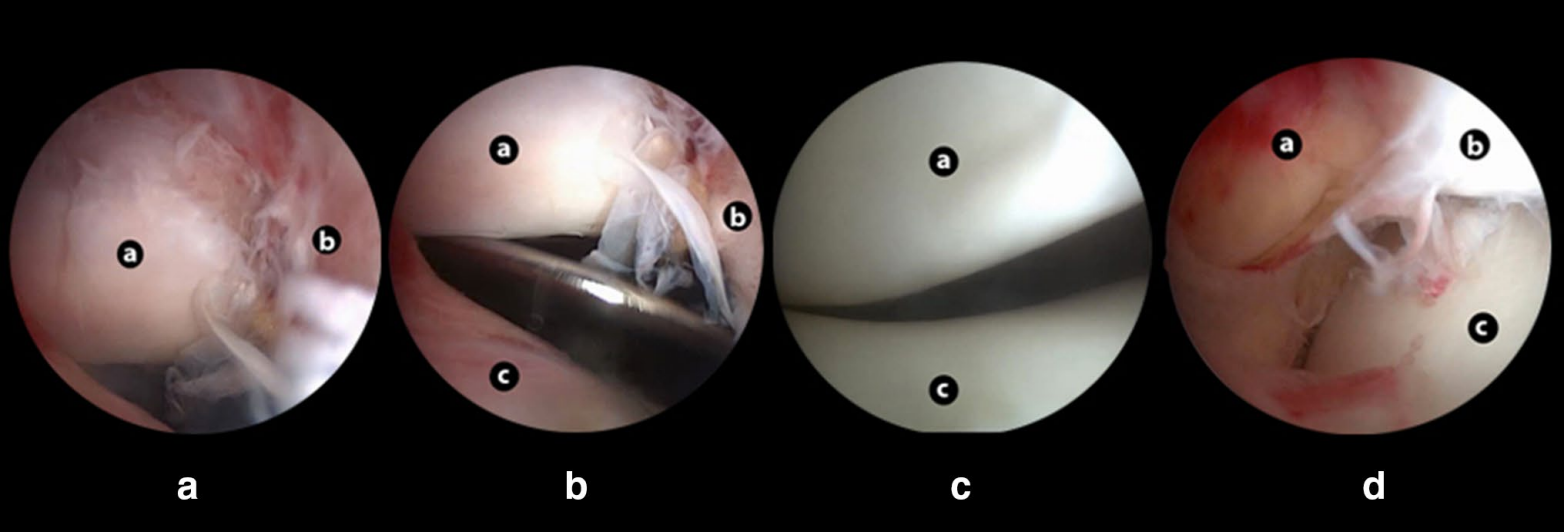
Arthroscopy images **a** laterally extra-articular dislocated patella folded in a pocket-like structure from the lateral retinaculum; **b** patellar reduction manoeuvre using a lever; **c** good patellar tracking after reduction; **d** damage of medial retinaculum structures. Anatomy references: a—patella; b—lateral retinaculum; c—femur; d—medial structures

Due to persistent irreducibility, a knee arthroscopy was performed in order to reduce the locked patellar dislocation. With the patient lying supine with a thigh tourniquet, standard anterolateral and anteromedial plus supra-lateral portals were performed with the knee in extension. Inflation of the knee joint with saline solution did not result reduction of the patellar dislocation. Arthroscopic inspection revealed a lateral extra-articular dislocated patella. The patella was folded in a pocket-like structure made from the lateral retinaculum that was hindering reduction. By applying lateral leverage with a tissue elevator the patella was relocated (like using a shoehorn). Further arthroscopic inspection revealed no other lesions within the joint except for the lesion of medial patellar retinaculum (Fig. 2).

Normal patellar tracking was confirmed arthroscopically. An extension splint was applied during 2 weeks allowing full weight bearing. Then a hinged brace was applied with weekly progressive flexion (30°–45°–60°–75°–90°) until complete ROM.

At final follow-up (6 months), the patient was able to return to all sport activities without limitations.

## Discussion

Lateral extra-articular locked patellar dislocation is a rare injury. Only 16 cases have been described so far [10]. It has been described that the patella locks laterally following an unusual mechanism that differs from classical imbalance of chronic lateral patellar instability.

A case of irreducible patella is mostly caused by a direct medial blow with the knee in extension or slight flexion [1, 4, 6, 9–11, 13, 14, 16, 22, 25]. It can also occur without direct trauma [2, 15, 17, 18, 21] and in patients with previous history of patellar dislocation as described by Higgins and Khalfaoui and Devgan et al. [5, 12].

The presented case is unique because the patella was locked due to soft tissue interposition. A tissue fold was created by the patella while moving laterally producing a button-hole mechanism that hindered relocation of the patella.

In this case no further imaging investigation was carried out beside initial radiological examination [1]. A good axial view was obtained and the authors considered that arthroscopy could help in defining other associated lesions.

Michels et al., Grewal et al., and Feibel et al., recommended the use of CT before open reduction [7, 10, 17]. It may help in localizing the patella and is a useful tool to rule out impaction fractures.

The reduction methods previously described for irreducible patella cases are either closed [1, 10, 13, 18, 22] by applying lateral pressure to the medial patellar surface while performing gentle knee hyperextension or by arthrotomy (mini-open approach) [2, 4–7, 9, 11, 12, 14–17, 21, 25] as opposed to arthroscopic as in the current case.

Closed reduction should always be the first step in uncomplicated patellar dislocations; however, in true irreducible cases arthroscopy or arthrotomy may be necessary in order to understand the blocking mechanism and to prevent additional damage to cartilage or soft tissue.

Considering the high incidence of cartilage damage, which can be as high as 94% of the cases in some series [3],^,^ a complete knee articular space inspection seems to be an advantage associated to the arthroscopic approach. Moreover, it allows an easy, reliable and dynamic method to confirm if the correct patellar tracking is achieved after reduction.

After patellar reduction is achieved, it is important to manage potential additional lesions present (e.g. bone fractures, cartilage or soft tissue injuries). With regard to injury of the medial structures, it has been well established in the literature that surgical repair does not improve the short- or long-term results in primary patellar dislocations when compared to conservative treatment [19]. As good patellar tracking was confirmed arthroscopically after patellar reduction and no major medial soft tissue injury was found, the standard conservative treatment protocol for primary patellar dislocations was used.

In current literature an immobilization period, with the knee in extension, ranging from 2 to 6 weeks post-operative was applied. In this case, a 2-week immobilization period followed by progressive rehabilitation with a semi-rigid knee brace was prescribed.

After the rehabilitation programme, the patient returned to sport activity without symptoms.

## Conclusion

Despite the major impact of the trauma mechanism, it shows that good outcome in the short- and mid-term can be expected.

To our knowledge, this is the first arthroscopic reduction method described for irreducible patellar dislocation representing a good alternative that allows for direct visualization of the reduction and complete articular inspection for additional injuries, being less invasive than the classic open arthrotomy.
